# Testing the effects on information use by older versus younger women of modality and narration style in a hospital report card

**DOI:** 10.1111/hex.13389

**Published:** 2021-12-24

**Authors:** Nida Gizem Yılmaz, Danielle R. M. Timmermans, Johanneke Portielje, Julia C. M. Van Weert, Olga C. Damman

**Affiliations:** ^1^ Department of Public and Occupational Health Amsterdam Public Health Research Institute, Amsterdam UMC, Vrije Universiteit Amsterdam Amsterdam The Netherlands; ^2^ Department of Communication Science Amsterdam School of Communication Research/ASCoR, University of Amsterdam Amsterdam The Netherlands; ^3^ Department of Medical Oncology Leiden University Medical Center Leiden The Netherlands

**Keywords:** audiovisual information, breast cancer, hospital report cards, information processing, modality, narration style, older women

## Abstract

**Background:**

Hospital report cards (HRCs) are usually presented in a textual and factual format, likely hampering information processing.

**Objective:**

This study aimed to investigate the effects of audiovisual and narrative information in HRCs on user responses, and to test differences between older and younger women.

**Design:**

A 2 (modality [textual vs. audiovisual]) × 3 (narration style [factual vs. process narrative vs. experience narrative]) online experiment was conducted. Information about breast cancer care was used as a case example. Age (younger [<65] vs. older [≥65]) was included as a potential effect modifier.

**Setting and Participants:**

A total of 631 disease‐naïve women (*M*_age_ = 56.06) completed an online survey. The outcomes were perceived cognitive load, satisfaction, comprehension, information recall and decisional conflict. Data were analysed using AN(C)OVAs.

**Results:**

Audiovisual (vs. textual) information resulted in higher information satisfaction across age groups, but was associated with lower comprehension in older women. An experience narrative (vs. factual information) increased satisfaction with attractiveness and emotional support of the information only in older women. A three‐way interaction effect was found, suggesting that older women were most satisfied with the comprehensibility of audiovisual factual or textual process narrative information. Younger women were most satisfied with the comprehensibility of audiovisual process narrative or textual factual information.

**Discussion and Conclusion:**

Audiovisual and narrative information in an HRC showed beneficial effects on satisfaction measures. In particular, audiovisual information could be incorporated into HRCs to increase satisfaction with information.

**Public Contribution:**

Lay persons helped in optimizing the visuals used in the stimulus materials by checking for clarity.

## 1 BACKGROUND

Hospital report cards (HRCs) are online decision support tools that can be used to compare and choose hospitals that best match patients' preferences concerning the services and quality provided.^1^, ^2^ Research shows that if HRCs are used, they are indeed an aid to choosing (or avoiding) particular hospitals.^3^ However, the use of HRCs generally remains low among patients.^4^, ^5^

Previous literature suggests several factors contributing to this low HRC uptake. One is that the content of HRCs is abstract and difficult to use when having to make multiattribute decisions, easily leading to cognitive overload.^6^, ^7^, ^8^, ^9^ For example, quality indicators in HRCs have been shown to be difficult to comprehend^10^ because they are usually described through large amounts of text^8^, ^9^ and fairly technical language.^6^ Moreover, quality indicators and hospital scores are often presented in a factual style. As a result, existing HRCs likely do not attract much interest and are cognitively burdensome,^1^, ^4^, ^8^, ^11^ hampering patients' motivation to engage with them.

To enhance information processing when provided with HRCs, one potentially fruitful strategy is to present information in an audiovisual modality (such as animated videos) instead of text. The assumed beneficial effect of audiovisual information is related to its ability to attract attention and foster interest in information.^12^, ^13^, ^14^ As proposed by the Cognitive Theory of Multimedia Learning,^15^ audiovisual information is known to induce a specific modality effect.^16^, ^17^, ^18^ This effect means that more information can be processed before cognitive overload occurs because verbal and visual information can be divided across multiple processing channels (i.e., both auditory and visual).^15^, ^19^ An audiovisual format—compared to text—has been associated with higher satisfaction with information,^12^ better comprehension^20^ and enhanced recall.^12^, ^13^, ^14^ As audiovisual modality is expected to contribute to better comprehension and information recall, it might also indirectly reduce patients' decisional conflict when provided with decision‐relevant information.^21^ Hence, the first hypothesis is: *Being provided with audiovisual information in a hospital report card, compared to textual information, will have a positive effect on satisfaction with information, information comprehension and information recall, and a negative effect on perceived cognitive load and decisional conflict*.

Another interesting presentation format is a narrative narration style instead of a factual narration style.^22^, ^23^, ^24^ Narratives are stories of other patients' experiences with a particular topic, in this case a healthcare choice.^25^, ^26^ Using narratives—compared to factual information—can support information processing by activating intuitive and deliberative reasoning simultaneously.^24^ It is known that patients often base their provider choice, at least partly, on anecdotal information (e.g., experiences of other patients; intuitive reasoning) and not solely on information from HRCs (deliberative reasoning).^22^, ^27^ This illustrates the potential of narrative information to enhance interest in and involvement with information,^22^, ^23^, ^24^, ^28^ a process called ‘immersion’.^23^, ^29^ The decision‐making literature further suggests that narratives can have elements that support patients specifically in information processing^23^ and decision‐making.^24^, ^30^, ^31^ Altogether, narratives can result in higher satisfaction with information,^12^ better comprehension,^32^ enhanced recall^12^, ^33^ and less decisional conflict.^34^ The second hypothesis is: *Being provided with narrative information in a hospital report card, compared to factual information, will have a positive effect on satisfaction with information, information comprehension and information recall, and a negative effect on perceived cognitive load and decisional conflict*.

However, the effectiveness of incorporating narratives into decision support tools generally remains unclear.^33^, ^35^, ^36^, ^37^ One explanation might be that not all types of narratives are equally beneficial in supporting information processing and decision‐making.^29^, ^38^, ^39^ A taxonomy of narratives in the field of decision‐making distinguishes three types of narratives.^38^ *Outcome narratives* contain information about the physical and psychological outcomes of decisions (e.g., what effects did a treatment have),^38^ and are hypothesized to cause direct changes in decisions. *Process narratives* focus on the cognitive axis of decisions (e.g., how to identify important decision dimensions),^38^ and are expected to positively contribute to information processing and knowledge. *Experience narratives* focus on the experiential axis of decisions (e.g., what visceral experiences and feelings did the diagnosis induce),^38^ and are hypothesized to influence affective forecasting and to increase knowledge. As an audiovisual modality and elements of process and experience narratives are both expected to positively impact information processing in HRCs, their combination was investigated in the current study. The third hypothesis is: *Being provided with audiovisual narrative information in a hospital report card, compared to other combinations of modality and narration style, will have a positive effect on satisfaction with information, information comprehension and information recall, and a negative effect on perceived cognitive load and decisional conflict*.

Testing the effects of modality and narration style is especially crucial in older adults such as older cancer patients, which is an ever‐growing group of patients worldwide.^40^ A previous study showed that especially older patients seem to be nonusers of HRCs.^5^ Older people are at risk of suboptimal information processing due to age‐related declines in working memory capacity and in the ability to process, comprehend and recall information.^20^, ^41^, ^42^, ^43^, ^44^, ^45^, ^46^, ^47^ The modality effect can thus be more paramount for older adults.^48^ Decision strategies are also known to change with age. It has been suggested that older adults rely more on intuitive and affective decision strategies.^49^, ^50^ Additionally, according to the Socio‐emotional Selectivity Theory, older adults' motivation to process information becomes more selective with age, resulting in an increased focus on emotionally meaningful information.^51^, ^52^, ^53^ This focus on intuitive reasoning and emotional information might lead to suboptimal decision‐making in older patients because deliberative strategies are used to a lesser extent.^49^, ^54^ Narratives might have the potential to compensate for this, when they are explicitly designed to induce both intuitive and deliberative information processing. Hence, the fourth hypothesis is: Older patients might benefit more from audiovisual and narrative (especially experience narrative) information (compared to textual and factual information, respectively), and their combination, than younger patients.

## 2 METHODS

### 2.1 Design

This study contained a between‐subjects factorial 2 (modality [text vs. audiovisual]) × 3 (narration style [factual vs. process narrative vs. experience narrative]) experimental design in which the manipulations were performed in the descriptions of quality indicators (i.e., the aspects on which the hospitals are compared) in an HRC. We chose to manipulate specifically this information because the indicators in fact form the basis of the hospital comparison and are used to choose a hospital.

Age (i.e., young [<65 years] vs. old [≥65 years]) was included as a potential effect modifier. Participants were stratified by age and randomly assigned to one of the six experimental conditions via automatic randomisation (allocation ratio = 1:1:1:1:1:1). The Medical Ethics Committee of Amsterdam UMC, location VUmc, approved the study (2016.587). The study was also preregistered (see https://osf.io/j5sp3?view_only=9a6f6f06d4024e498ce9c7d940ecc193). Written consent was obtained from the participants.

### 2.2 Materials

**Figure 1:**
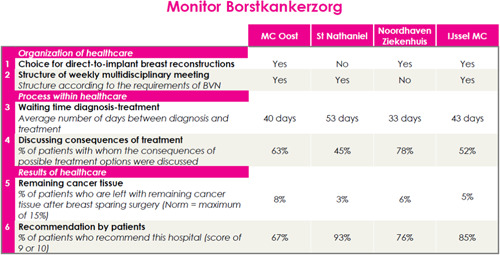
Fictitious hospital report card used as stimulus material

All provided information contained fictitious but realistic information in an HRC (i.e., based on a real Dutch HRC for breast cancer patients; https://borstkanker.nl/nl/monitor-borstkankerzorg-0) about four hospitals (*MC Oost, St Nathaniel, Noordhaven Ziekenhuis* and *IJssel MC*) and six quality indicators (Figure 1). Before data collection, three textual scripts were developed: (1) factual; (2) process narrative; and (3) experience narrative. The first script contained factual information and started with information about the aim of the HRC. Next, information was provided about the process of evaluating hospitals before making a choice, and explained the three categories of quality indicators registered for Dutch hospitals (i.e., ‘organization of healthcare’, ‘process within healthcare’, and ‘results of healthcare’).

The second and third scripts contained exactly the same information as the factual version, but were written in a narrative style. Hence, in all scripts, the basic content was identical. For the second script, information was enriched with contextual information, turning the script into a process narrative,^38^ by letting a female character diagnosed with breast cancer tell her story about how she chose a hospital. The process narrative concentrated on the cognitive process of comparing hospitals and weighing quality indicators, as would be characteristic of a normative decision‐making model^38^ (red text Appendix SA).

The third script elaborated on the exact same information as in the second script. This script was written as an experience narrative.^38^ In addition to cognitive information, experiential (including emotional) context concerning the process of choosing a hospital was added (blue text Appendix SA). The experience narrative was expected to be perceived as ‘more emotional’ than the process narrative, based on a previous study that investigated the effects of process narratives compared to experience narratives on treatment decision‐making in breast cancer care. This study concluded that experience narratives were associated with a greater ability to imagine experiences with treatment.^55^

The scripts were the basis of the textual and audiovisual conditions. In the textual conditions, participants received one of the scripts (i.e., either factual, process narrative or experience narrative). For the audiovisual conditions, the three textual scripts were recorded as voice‐overs (female voice) and used in animated videos (i.e., ‘simulated motion picture depicting the movement of drawn [or simulated] objects’).^56^ For the animated videos, 16 visuals depicting information elements from the scripts were pilot‐tested among five disease‐naïve women aged 65 years or older. Based on this pilot, 11 visuals were finalized and used in the animations. Ultimately, three animations were developed: (1) factual information; (2) process narrative information; and (3) experience narrative information. A link to the animations, in Dutch, can be found in Appendix SB.

### 2.3 Participants

Participants were women aged 18 years and older who had no breast cancer or a history of having it (i.e., disease‐naïve). We chose to recruit so‐called analogue patients to avoid participants having prior knowledge about the quality indicators. Using analogue patients has been shown to be a valid approach in experimental communication research.^57^ Participants were included if they had sufficient mastery in both reading and speaking Dutch. Participants were recruited through an online research panel called Flycatcher Panel, which is ISO20252‐ and 26362‐certified. An a priori sample size calculation in G*Power for a 2 × 3 factorial design with a small to medium effect size of 0.20 (Cohen's *f*) and a two‐sided significance level of .05 showed that at least 619 participants needed to be included for sound power (0.95). Ultimately, 631 participants were included.

### 2.4 Procedure

Flycatcher sent participants a link to the online survey. Participants were first informed about the aim and content of the study, the confidentiality of data and voluntary participation. Next, participants provided informed consent. Participants were randomly assigned to one of the six conditions. After reading/watching the information provided, participants were directed to the survey. Participants had to fill in all questions on a page before they could move on to the next page. This ensured that only completed surveys were submitted and no data were missing. For examining the data quality, Flycatcher checked completed surveys on answers to open questions (e.g., information recall), consistency in answers, straight lining and time spent on completing the survey.

### 2.5 Measures

The survey covered our dependent variables (see below). Moreover, sociodemographic and medical background variables (i.e., age, educational level, comorbidity, quality of life and diagnosis), and control variables (i.e., transportation and identification) were included. Comorbidity was defined as having two or more health problems, and quality of life was measured by two items (i.e., *How would you rate your overall health during the past week?* and *How would you rate your overall quality of life during the past week?*).^58^ Health literacy and numeracy were included in additional analyses as covariates because both variables are known to be strongly related to information processing. Health literacy was measured by the Newest Vital Sign‐D,^59^ containing six questions. Answering four or more questions correctly was considered as ‘adequate’ health literacy.^59^ Numeracy was measured by the single‐item Berlin Numeracy Test,^60^ and answering the question correctly was considered as ‘adequate’ numeracy. Transportation (i.e., being cognitively, emotionally and imaginarily involved in the text/video) was measured by eight items (e.g., *I wanted to know how the story from the texts/videos ended*; *α* = .73).^61^ Identification was measured by three items (e.g., *In my imagination, it was like I was [character in stimulus material]*; *α* = .95).^62^ All items consisted of a 7‐point Likert scale (*1* = *totally disagree* to *7* = *totally agree*).

### 2.6 Perceived cognitive load

Four items measured on a 7‐point Likert scale (*1* = *strongly disagree* to *7* = *strongly agree*) developed by Eveland and Dunwoody^63^ were used to measure perceived cognitive load (*α* = .82).

### 2.7 Satisfaction with the information

Twelve items measured on a 7‐point Likert scale (*1* = *totally disagree* to *7* = *totally agree*) from the Website Satisfaction Scale were used to measure satisfaction with information (*α* = .94).^64^ Both a total scale score and scores for three subscales were calculated. Three items related to the subscale ‘Satisfaction with attractiveness’ (*α* = .89), five items related to the subscale ‘Satisfaction with comprehensibility’ (*α* = .92) and four items related to the subscale ‘Satisfaction with emotional support’ (*α* = .95).

### 2.8 Information comprehension

Information comprehension was measured by fifteen multiple‐choice questions.^65^ An example of an information comprehension question was as follows: *‘For Nina, it is not important that she can receive a direct‐to‐breast implant after surgery. Which hospital would be the best choice for her?’* Answer options for this question were as follows: *(a) MC Oost, (b) St Nathaniel, (c) Noordhaven Ziekenhuis, (d) IJssel MC, (e) It does not matter* and *(f) I don't know*. For each question, one or two response options could be correct. Participants were enabled to revisit the stimulus materials while filling in comprehension questions. The final score was the sum of correct answers, and ranged from 0 to 15.

### 2.9 Information recall

Fourteen open‐ended questions based on the Netherlands Patient Information Recall Questionnaire were used to measure information recall.^43^ All questions related to the information about quality indicators (see Appendix SC for the recall questions). Participants were not able to revisit the stimulus materials while answering the questions. Before data analysis, a preliminary codebook, including scores for correct answers, was developed by the researchers (N. G. Y. and O. C. D.). This codebook was used by the two researchers to independently score 5 of the 14 questions (35.7%). Agreement ranged from 60.0% to 79.2%. After the first round, the researchers discussed their scores, and adapted the codebook accordingly. The adapted codebook was used by the researcher (N. G. Y.) to rescore the answers. To ensure the validity of the scores, the two researchers discussed the new scores for a second time. Agreement then ranged from 85.5% to 98.0%. At the end of this iterative process, the final codebook was developed, and one researcher (N. G. Y.) went through all the answers again. The maximum score for a correct answer differed per question, and ranged from 0 to 2 points. Sum scores ranged from 0 to 18.

### 2.10 Decisional conflict

Sixteen items measured on a 5‐point Likert scale from the Decisional Conflict Scale^66^ were used (*α* = .94). Both a total scale score and scores for five subscales were calculated. Three items related to ‘Informed’ (*α* = .86), three items related to ‘Values clarity’ (*α* = .87), three items related to ‘Support’ (*α* = .74), three items related to ‘Uncertainty’ (*α* = .87) and four items related to ‘Effective decision’ (*α* = .91).

### 2.11 Pilot tests for the development of stimulus materials

Before data collection, a pilot test of the stimulus materials and survey was conducted. For the textual conditions, the scripts were pretested among 42 women (*M*_age_ = 60.95). These women were recruited through PanelCom (http://www.panelcom.nl), and were randomly assigned to the factual (*n* = 14), process narrative (*n* = 14) or experience narrative (*n* = 14) textual script. The two narrative texts were perceived as more narrative compared to the factual text (*p* < .001). Using factor analysis, three subscales were constructed for use as a manipulation check in the survey, with three items belonging to the subscale ‘Factual’ (*α* = .84), three items belonging to ‘Process narrative’ (*α* = .93) and three items belonging to ‘Experience narrative’ (*α* = .92). All items were measured on a 7‐point Likert scale (*1* = *totally disagree* to *7* = *totally agree*).

### 2.12 Manipulation check

Factual information was perceived as more factual (*M* = 15.31, SD = 3.06) than process narrative information (*M* = 13.27, SD = 3.60), *t*(404) = 6.12, *p* < .001, 95% confidence interval (CI): [1.38, 2.69]. Process narrative information was perceived as more narrative (*M* = 14.90, SD = 4.12) than factual information (*M* = 9.58, SD = 4.19), *t*(404) = −12.90, *p* < .001, 95% CI: [−6.13, −4.51]. Hence, it can be concluded that the manipulation ‘factual vs. process narrative’ was successful. Factual information was also perceived as more factual (*M* = 15.31, SD = 3.06) than experience narrative information (*M* = 12.65, SD = 3.57), *t*(423) = 8.19, *p* < .001, 95% CI: [2.02, 3.29]. Furthermore, experience narrative information was perceived as more narrative (*M* = 14.70, SD = 3.93) than factual information (*M* = 9.23, SD = 4.29), *t*(423) = −13.74, *p* < .001, 95% CI: [−6.26, −4.69]. Hence, it can be concluded that the manipulation ‘factual vs. experience narrative’ was successful. Process narrative information was perceived as less narrative (*M* = 14.90, SD = 4.12) than experience narrative information (*M* = 15.96, SD = 3.74), *t*(429) = −2.79, *p* = .005, 95% CI: [−1.80, −0.31]. Additionally, experience narrative information was perceived as more narrative (*M* = 14.70, *SD* = 3.93) than process narrative information (*M* = 12.84, SD = 4.22), *t*(429) = −4.73, *p* < .001, 95% CI: [−2.63, −1.09]. Hence, it can be concluded that the manipulation ‘process narrative vs. experience narrative’ was successful.

### 2.13 Statistical analyses

Data analysis was conducted using SPSS, version 26. Differences between conditions and differences between younger and older women in the control variables (i.e., health literacy, numeracy, transportation, identification) and the background variables (i.e., age, level of education, comorbidity, quality of life and diagnosis) were tested using one‐way analysis of variances (ANOVAs). The effects of modality, modality*age, narration style, narration style*age, modality*narration style and modality*narration style*age on the dependent variables were tested using two‐way ANOVAs. Post‐hoc analyses were performed to analyse the differences between the conditions. We adopted a cut‐off age of 65 years to categorize participants into ‘younger’ (18–64 years old) and ‘older’ (65 years or older) participants, which is generally accepted in studies that investigate the effects of ageing on health‐related outcomes. To adjust for the effects of multiple‐hypothesis testing, a Bonferroni correction was applied.

Descriptive statistics showed that younger women had a higher level of eduaction, and had higher health literacy and numeracy than older women (see Section Design, 3 and Table 1). In analysing the interaction effects of the manipulations with age (RQ1b, RQ2b and RQ3b), we took into account level of education, health literacy and numeracy as confounders in additional analysis of covariances (ANCOVAs). As the three variables were highly correlated, three separate ANCOVAs were conducted. All findings with *p* ≤ .05 were considered significant.

**Table 1:** Sample characteristics

	Total sample (*N* = 631)	Younger patients (*n* = 334)	Older patients (*n* = 297)
Sample characteristics			
Age (*M* ± SD)	56.06 ± 16.43	43.60 ± 12.63	70.07 ± 4.77**
Level of education (*n*, %)			
Low	188 (29.8)	56 (16.8)	132 (44.4)**
Moderate	266 (42.2)	174 (52.1)	92 (31.0)
High	177 (28.1)	104 (31.1)	73 (24.6)
Comorbidity (% yes)	72.6	70.7	74.7
Quality of life (*M* ± SD; range = 4–14)	10.76 ± 2.26	10.89 ± 2.17	10.61 ± 2.36
Diagnosis (% yes)			
Lung	0.5	0.0	0.9
Colorectal	0.6	0.3	1.0
Gynaecological	1.1	0.6	1.7
Urological	0.2	0.0	0.3
Skin	1.7	0.9	2.7
Other	1.3	0.8	2.4
Control variables			
Health literacy (*M* ± SD; range = 1–6)	4.84 ± 1.53	5.30 ± 1.22	4.32 ± 1.67**
Numeracy (% correct)	37.3	49.3	23.0**
Transportation (*M* ± SD; range = 12–53)	33.58 ± 7.04	33.68 ± 7.26	33.47 ± 6.79
Identification (*M* ± SD; range = 3–21)	8.93 ± 4.48	8.62 ± 4.60	9.29 ± 4.31
Outcome measures			
Perceived cognitive load (*M* ± SD; range = 4–28)	13.38 ± 4.75	12.49 ± 4.65	14.38 ± 4.66**
Decisional conflict			
Low (*%*)	13.8	11.7	16.2
Moderate (*%*)	29.8	29.9	29.6
High (*%*)	56.4	58.4	54.2
Comprehension of information (*M* ± SD; range = 0–15)	10.88 ± 4.20	12.15 ± 3.34	9.45 ± 4.60**
Information recall (*M* ± SD; range = 0–18)	3.63 ± 3.11	4.35 ± 3.28	2.81 ± 2.70**
Satisfaction with information (*M* ± SD)			
Attractiveness (range = 3–21)	12.11 ± 3.74	12.09 ± 3.64	12.13 ± 3.86
Comprehensibility (range = 5–35)	25.26 ± 5.51	25.72 ± 5.39	24.76 ± 5.71*
Emotional support (range = 4–28)	15.14 ± 5.55	14.94 ± 5.32	15.36 ± 5.79

** *p* < .001.

* *p* ≤ .05.

## 3 RESULTS

### 3.1 Sample characteristics

Table 1 presents the sample characteristics. In the final sample, women were aged between 19 and 95 years. On average, participants rated their quality of life as moderate (*M* = 10.76, SD = 2.26; range = 4–14). The majority of all participants showed an adequate level of health literacy (81.3%), but less than half of them (37.3%) answered the numeracy question correctly. Transportation into the story was moderate (*M* = 33.58, SD = 7.04; range = 12–53), while identification with the character from the narratives was quite low (*M* = 8.93, SD = 4.48; range = 3–21). There were no differences between older and younger participants in transportation and identification.

Table 2 presents the mean scores on the control and outcome measures per experimental condition. No significant differences existed between conditions in control variables, and outcomes measures, except for overall satisfaction with information and satisfaction with the attractiveness of information. For readability purposes, the *F*‐test statistics per research question can be found in Appendix SD.

**Table 2:** Mean ± standard deviation per control and outcome measure and condition

	Textual, factual (*n* = 105)	Textual, process narrative (*n* = 106)	Textual, experience narrative (*n* = 114)	Audiovisual, factual (*n* = 95)	Audiovisual, process narrative (*n* = 100)	Audiovisual, experience narrative (*n* = 111)
Control variables						
Health literacy	4.83 ± 1.55	4.94 ± 1.43	4.82 ± 1.55	4.77 ± 1.59	4.78 ± 1.61	4.86 ± 1.56
Numeracy	0.32 ± 0.47	0.37 ± 0.48	0.41 ± 0.49	0.29 ± 0.46	0.44 ± 0.50	0.40 ± 0.49
Transportation	33.07 ± 6.08	32.78 ± 8.00	33.91 ± 6.58	34.13 ± 6.21	33.87 ± 7.64	33.77 ± 7.49
Identification	a	7.92 ± 4.07	9.20 ± 4.82	a	9.21 ± 4.44	9.36 ± 4.44
Outcome measures						
Perceived cognitive load	14.28 ± 4.52	13.23 ± 4.82	13.30 ± 5.09	13.14 ± 5.08	13.24 ± 4.94	13.09 ± 4.02
Satisfaction with information						
Total	49.23 ± 10.58^b^, ^c^	51.39 ± 12.86	51.90 ± 13.05	54.58 ± 12.57^b^	53.41 ± 13.79	54.75 ± 13.29^c^
Attractiveness	19.73 ± 4.92^d^, ^e^, ^f^	20.85 ± 5.82	21.28 ± 5.64	22.91 ± 5.64^d^	22.39 ± 6.10^e^	22.45 ± 6.19^f^
Comprehensibility	15.28 ± 2.90	15.80 ± 3.30	15.61 ± 3.63	15.86 ± 3.62	15.85 ± 3.85	16.39 ± 2.97
Emotional support	14.22 ± 5.01	14.74 ± 5.40	15.01 ± 5.79	15.81 ± 5.46	15.17 ± 5.56	15.91 ± 5.92
Information comprehension	10.90 ± 4.00	11.42 ± 3.83	11.30 ± 3.75	10.02 ± 4.89	10.55 ± 4.60	10.95 ± 4.08
Information recall	3.70 ± 2.97	3.45 ± 3.14	3.74 ± 3.08	3.22 ± 3.07	4.00 ± 3.51	3.61 ± 2.93
Decisional conflict						
Total	42.89 ± 18.91	44.43 ± 22.04	45.51 ± 20.20	42.25 ± 16.94	44.48 ± 19.88	40.18 ± 19.41
Informed	37.14 ± 17.30	37.89 ± 21.34	36.92 ± 18.25	37.19 ± 14.68	37.58 ± 18.93	33.03 ± 18.12
Values clarity	33.17 ± 19.16	31.60 ± 19.71	33.26 ± 19.09	31.93 ± 18.06	32.58 ± 19.28	30.78 ± 18.66
Support	30.71 ± 18.43	30.90 ± 20.02	33.63 ± 18.97	30.53 ± 17.00	31.92 ± 18.73	31.38 ± 19.53
Uncertainty	41.35 ± 21.46	44.97 ± 22.94	44.59 ± 22.77	39.47 ± 18.12	44.00 ± 21.36	38.36 ± 20.79
Effective decision	32.62 ± 17.29	35.38 ± 19.65	36.62 ± 19.85	32.96 ± 16.27	35.00 ± 17.88	30.41 ± 16.84

^a^ Identification was measured only in the narrative conditions because it was only in these conditions that a character was telling a story.

^b^ *M*_dif_ = −5.35, *p* = .047.

^c^ *M*_dif_ = −5.52, *p* = .023.

^d^ *M*_dif_ = −3.17, *p* = .002.

^e^ *M*_dif_ = −2.66, *p* = .015.

^f^ *M*_dif_ = −2.72, *p* = .008.

### 3.2 Effects of modality and interaction between modality and age

We found a significant main effect of modality on four out of the 13 outcome measures. Women receiving audiovisual information were more satisfied with information in general (*M*_dif_ = 3.39, *p* = .001, 95% CI: [1.40, 5.38]), and particularly with the attractiveness of information (*M*_dif_ = 1.93, *p* < .001, 95% CI: [1.03, 2.83]) and emotional support from information (*M*_dif_ = 0.97, *p* = .028, 95% CI: [0.11, 1.84]) than women receiving textual information. We observed the reverse effect for comprehension, with women receiving audiovisual information comprehending information less (*M*_dif_ = −0.68, *p* = .042, 95% CI: [−1.34, −0.03]).

A significant interaction between modality and age was found, showing that older women receiving textual information, compared to older women receiving audiovisual information, had significantly higher comprehension (*M*_dif_ = 1.44, *p* = .002, 95% CI: [0.53, 2.34]). Among younger women, no interaction effects were found. After adjusting for educational level or health literacy in ANCOVAs, there was no longer any interaction between modality and age for comprehension. In contrast, after adjusting for numeracy, the interaction effect remained intact.

### 3.3 Effects of narration style and interaction between narration style and age

No significant main effects of narration style were found on any of our 13 outcome measures. However, significant interaction effects were found between narration style and age. In older women, experience narrative information, compared to factual information, was associated with higher overall satisfaction with information (*M*_dif_ = 4.95, *p* = .018, 95% CI: [0.65, 9.25]), and specifically higher satisfaction with emotional support from information (*M*_dif_ = 2.11, *p* = .020, 95% CI: [0.25, 3.97]). In contrast, among younger women, no significant differences were found. Furthermore, older women receiving experience narrative information, compared to younger women receiving experience narrative information, were significantly more satisfied with emotional support from information (*M*_dif_ = 2.22, *p* = .003, 95% CI: [0.77, 3.67]). After adjusting for educational level or health literacy in two additional ANCOVAs, interaction effects remained intact. After adjusting for numeracy, the interaction effect between narration style and age on satisfaction with emotional support from information remained intact, but the effect on overall satisfaction with information was no longer significant.

### 3.4 Interaction effects of modality and narration style, and between modality, narration style and age

**Figure 2:**
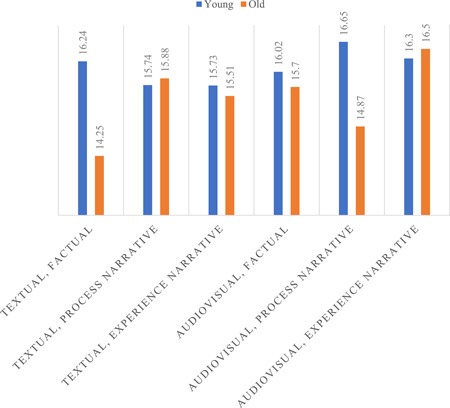
Interaction effect of modality * narration style * age on satisfaction with the comprehensibility of information (range = 5–35; *p* = .025)

No significant two‐way interactions between modality and narration style were demonstrated. We did find a significant three‐way interaction for satisfaction with comprehensibility of information (see Figure 2), but not on the other outcome measures. Older women receiving audiovisual factual information, compared to older women receiving textual factual information, were more satisfied with the comprehensibility of information (*M*_dif_ = 1.44, *p* = .035, 95% CI: [0.10, 2.78]). The same was found for older women receiving textual process narrative information compared to textual factual information (*M*_dif_ = 1.62, *p* = .050, 95% CI: [−0.00, 3.24]). These effects were not found for younger women. Younger women receiving audiovisual process narrative information (*M*_dif_ = 1.79, *p* = .008, 95% CI: [0.46, 3.11]) or textual factual information (*M*_dif_ = 1.99, *p* = .003, 95% CI: [0.70, 3.27]), compared to older women receiving the same information, were more satisfied with the comprehensibility of information. After adjusting for educational level, health literacy or numeracy in an additional ANCOVA in three separate models, the interaction effect on satisfaction with comprehensibility remained intact. After adjusting for numeracy, the interaction effect on overall satisfaction with information and on making informed choices (subscale of decisional conflict) became significant.

## 4 DISCUSSION

This study aimed to investigate the effects of audiovisual and narrative information in HRCs on user responses on breast cancer care, and to test differences between older and younger respondents. The results showed that audiovisual information resulted in a positive effect on information satisfaction measures across age groups. In older women, audiovisual information was also associated with lower information comprehension as compared to text. The detrimental effect of audiovisual information on comprehension in older women appeared, at least for a great part, to be attributable to older women's lower educational level/health literacy. For narration style, no significant main effects were found on women's responses to the information. However, interaction effects suggested that information in an experience narrative was especially beneficial for older women, while for younger women, the narration style did not play a huge role. The beneficial effect of experience narrative information in older women appeared, at least partly, to be attributable to their lower numeracy levels. Finally, the three‐way interaction effect for satisfaction with comprehensibility of information suggested that older women were especially supported by audiovisual factual information or by textual process narrative information. In contrast, for younger women, the audiovisual process narrative information and the textual factual information increased satisfaction with comprehensibility.

In terms of satisfaction with several aspects of the information, audiovisual information seemed to have benefits over textual information. However, this effect did not translate into better information processing in terms of, for instance, perceived cognitive load, comprehension or recall. In older women with lower educational level or health literacy, audiovisual information even led to lower comprehension. Hence, although audiovisual information seems promising in terms of increasing satisfaction with HRC information, the conditions under which it would also be effective for information processing outcomes should be investigated. Another point to consider is how HRC content should be formed. In our study, an existing HRC was highly simplified, but it still contained a rather classic format and percentages to express numerical information. Numerical formats should ideally be accompanied by graphical or evaluative presentation.^7^ Additionally, we did not check whether the language in the simplified HRC was at the B1 level. The negative effect of audiovisual information on comprehension might mean that our HRC content was still too difficult to process (both content‐wise and language‐wise), even when presented in audiovisual modality. Moreover, in the Netherlands, the costs of oncological care are covered by insurance companies. As such, costs were not an indicator in our HRC. In many other countries, however, costs might be an important indicator to weight in the decision‐making process. Another point to consider is the user options of the HRC. From the field of health literacy, we know that people with lower health literacy in general need more time to process information.^67^ Being allowed to absorb information at one's own pace (i.e., self‐pacing), including audiovisual materials, has been shown to contribute to higher comprehension in other studies.^14^, ^68^ In our study, women were not able to self‐pace the absorption of information in the audiovisual materials, which may be problematic for those who might need extra time. Altogether, the negative effect of audiovisual information on information comprehension might indicate that HRC content could be further adjusted by, for instance, including graphical formats,^10^, ^69^, ^70^ or providing self‐pacing possibilities. A third point should be kept in mind, however: using audiovisual modality also has some practical downsides. For instance, audiovisual information, or more specifically the visuals accompanying the relevant parts of the text, cannot be easily provided in a leaflet. In terms of costs, developing audiovisual material is also more expensive than developing text.

Our second hypothesis is related to the effectiveness of narrative information in enhancing information evaluation and processing. The Narrative Immersion Model postulates that interest should be captured first and engagement should be induced before the effect of narratives, that is, immersion, occurs.^29^ The overall lack of effects of narration style in our study, combined with the finding that narrative information did not result in higher transportation or identification (see Table 2), might mean that narration style does not induce the transportation and immersion for HRCs. Hence, the factual information might have been adequate to make sense of the importance of using HRCs. The results might have been different in an actual patient population because hospital choice is then actually more relevant.

Despite a lack of overall effects of narration style, it is interesting that including experiential/emotional contextual information in materials induced higher satisfaction with emotional support in older women. Older adults, in general, are assumed to be more in need of emotional support in information provision.^51^, ^54^ As such, providing older women with experience narrative information might enhance their ability to imagine experiences with choices, in turn fulfilling their need for emotional support. However, this effect did not translate into beneficial effects on information comprehension or information recall, as expected based on the Socio‐emotional Selectivity Theory and a previous study.^52^, ^53^, ^64^ A possible explanation lies in the information type/form that we provided. A previous study focused on adding static illustrations to textual health information about treatment showed that when illustrations were added, older adults were more satisfied with emotional support from information than younger adults, and that this resulted in better recall.^64^ It should be kept in mind that treatment and hospital choice are different types of choices. Moreover, the overall quality of care is high in the Netherlands, which might lead to less interest in HRCs.

Our study has some limitations. First, due to the inherent characteristics of narratives, the narrative conditions (and especially the experience narrative) contained more information than the factual condition. We intentionally chose to keep the basic information identical in our experimental conditions and to add contextual information to the narrative conditions. A limitation is, however, that the difference in length might partly explain why narratives did not produce enhanced cognitive outcomes. It has been shown before that providing more information can lead to less recall.^71^ Older adults are especially known to experience difficulties in distinguishing between main and side issues.^20^ Keeping the basic content identical can, however, also be perceived as a strength of the study because effects found can only be attributed to the information type, and not to differences in basic content. Nevertheless, this limitation calls for future research into the effects of narratives. For example, should the length of narratives be kept identical and the content be different, or should the content (at least the basic content) be identical and the length be different? Second, we adopted a cut‐off age of 65 years. This cut‐off is often used in health‐related studies to investigate the effects of ageing.^72^ Nevertheless, such cut‐offs always remain arbitrary, and audiovisual and narrative information might have different effects on information processing in the oldest‐old population. In our sample, however, adopting a cut‐off of 70 years did not result in findings other than the ones reported. Third, we tested multiple hypotheses. However, by applying the Bonferroni correction, we adjusted for the effects of multiple‐hypothesis testing. Finally, recruitment of participants who had no breast cancer might have led to a sample of relatively less motivated participants to process the information. Hence, HRC users from the actual target population might be more motivated. Also, adjusting for (especially) health literacy and numeracy—variables that are highly correlated with educational level—might mean that we were overcorrecting.

## 5 CONCLUSION

Our study mainly yielded beneficial effects of audiovisual and narrative information on satisfaction measures, which did not translate into differences in information processing (e.g., information comprehension and information recall). We can recommend incorporating audiovisual information in HRCs. An audiovisual format seems to increase satisfaction with the information. Higher satisfaction with the information, in turn, might lead to a higher uptake of HRCs, ultimately contributing to better informed hospital choices. However, given the limited effects on the other study outcomes, it should be investigated whether and when adding audiovisual information to HRCs is of real added value in practice. Narrative information cannot necessarily be recommended for broad groups of patients, but the effects of narratives in older women suggest that this strategy might have potential. We recommend elaborating on the effects of experience narratives in different groups of older patients in future research.

## CONFLICT OF INTERESTS

The authors declare that there are no conflict of interests.

## AUTHOR CONTRIBUTIONS

Nida Gizem Yılmaz contributed to the design of the work, analysis and interpretation of data, and writing of the manuscript. Danielle R. M. Timmermans contributed to the acquisition, design of the work, interpretation of data and giving feedback on the manuscript. Julia C. M. Van Weert contributed to the acquisition, design of the work, interpretation of data and giving feedback on the manuscript. Johanneke Portielje contributed to the interpretation of data and giving feedback on the manuscript. Olga C. Damman contributed to the acquisition, design of the work, analysis and interpretation of data, and giving feedback on the manuscript.

## Supporting information

Supplementary information.

Supplementary information.

Supplementary information.

Supplementary information.

## Data Availability

The data that support the findings of this study are available from the corresponding author upon reasonable request.
