# Supplementary material for: Testing the effects on information use by older versus younger women of modality and narration style in a hospital report card
Source: Health Expect. 2021 Dec 24;25(2):567–78. doi: 10.1111/hex.13389 (PMC8957735; doi:10.1111/hex.13389)
Supplement: Supplementary file 1 — Supplementary information. [file HEX-25--s004.pdf]

Appendix A. *Script used as stimulus materials.*

You will listen to the story of Marloes. Marloes is 50 years old and was diagnosed with breast cancer 1 year ago. Marloes is telling about choosing a hospital.

When I was diagnosed with breast cancer, I quickly had to choose a hospital where treatment would take place. That was not easy for me, because I had a busy life with my children living at home and my job. And I was still frightened by the fact that I had been diagnosed with breast cancer; I hadn't seen that coming at all. At first, I wanted to choose the hospital nearest by home. That would be most convenient for my busy life. However, later on, I realized that several aspects were important for my choice.

First, I received information from my doctor and from people I know. In the beginning, I liked that, because they all thought very much along with me and of course wanted the best for me. But it was also important for me to be in control when making my choice. It concerned my health, namely, and we were not speaking about a flu. And, well, the Monitor Borstkankerzorg helped me a lot. The purpose of the Monitor Borstkankerzorg is to help patients choose a hospital where research into breast abnormalities and possible treatment will take place. It was not possible for me to research each hospital by myself, of course, that is just too much. Therefore, it was fine that to be able to look into information about hospitals in the Monitor Borstkankerzorg.

Well, so I wanted to think carefully about my hospital choice. That was quite stressful in the beginning. After the diagnosis, I did not know what everything means at once. What I did know, was that I had to think about what I personally find important in my care, and that I subsequently

searched for a hospital that meets my wishes. I knew, of course, that the quality of care in Dutch hospitals is good, but even so you sometimes hear that hospitals differ at some points. Ultimately, I looked at three types of information about the quality of care. The first one concerned the organization of care, the second one concerned the care process, and the third one concerned the result of the care provided.

Information about the organization of care concerns how the hospital has organized care in order to offer responsible care. For example, do you think it is important that in a hospital it is possible that the doctors immediately make a new breast when the breast is removed? Or do you value that the composition of the weekly Multidisciplinary Consultation, abbreviated as MDO, complies with the guidelines of the Dutch Breast Cancer Association? Then, information about the organization of care is particularly important for you. The MDO, moreover, is a consultation moment between different doctors, such as oncologists, surgeons, and radiologists, but also nurses, who are all involved in the treatment. For me, both things were important: that my new breast could be made immediately when my breast had been removed, and that the MDO was composed according to the guidelines of the Breast Cancer Association. It is, I think, very personal for a patient whether you think it is important that the new breast can be implanted immediately. I was taken up by the thought of waking up without breasts after surgery. So, it was very nice that this could be done immediately. The MDO gave me, as a patient, the feeling that the best option for me was being judged from different angles. I am quite afraid of side effects from all kinds of treatments. So, I wanted to entrust that the doctors would discuss the treatment plan with each other in view of those side effects. That is a reassurance that I badly needed as a patient.

Information about how the care process proceeds concerns how certain processes work in a hospital. Do you find it important that a hospital has short waiting times? Or that the consequences of certain treatments are being discussed with the patient? Then, information about the care process is particularly important for you. For me, the waiting times were important, but what was even more important was that the consequences of a certain treatment were being discussed. I noticed that I really got into a rollercoaster after I was diagnosed with breast cancer. Everything went very fast. I heard I had breast cancer and a week later I sat on the couch with drains next to me. These are thin tubes that drain blood and fluid from the wound. This allows your wound to heal better. I deliberately chose for a hospital with short waiting times, but in hindsight it might not have been a problem if I had a little more time to make a choice. "Get rid of that mess, quickly out of my body." That is what I first thought, because I was just very afraid of the disease, but also because I wanted to work on my recovery quickly. I wanted to be the strong mother again for my children. I now know that those few days don't matter much. Therefore, now I say: it is also important for me that the consequences of a certain treatment, such as long-term fatigue what many patients suffer from, are being discussed appropriately. My doctor had talked little about that, but I did notice that I was experiencing side effects, such as severe fatigue, that I was so afraid of. I was sorry, yes, that not so much attention was paid to that. I called the doctor a few times later because I was concerned, the fatigue lasted quite a long time namely.

Information about the result of the care provided is what the result of the care is. For example, do you think it is important that no cancer tissue is left behind after surgery? Dutch hospitals, moreover, meet established standards for this. Or do you think it is important that patients are satisfied with the hospital? Then, information about the result of the care provided is particularly important. For me personally, it was very important in how many patients cancer

tissue was left behind after surgery. If cancer tissue remains, you’re screwed again. So, for me it was important to evaluate that carefully. I know someone to whom that happened. At that time, cancer tissue had remained and unfortunately, she had to go to the hospital again. That thought frightened me. Luckily, Dutch hospitals meet the norms that are set for remaining cancer tissue.

Altogether, it is quite difficult, such a hospital choice. Especially when you have to make this choice for the first time. You do not know, namely, what information is important for you. The Monitor Borstkankerzorg provides a good summary of the information, and, hence, has helped me.
