# Supplementary material for: Testing the effects on information use by older versus younger women of modality and narration style in a hospital report card
Source: Health Expect. 2021 Dec 24;25(2):567–78. doi: 10.1111/hex.13389 (PMC8957735; doi:10.1111/hex.13389)
Supplement: Supplementary file 2 — Supplementary information. [file HEX-25--s003.pdf]

Appendix B. *Animations used in the study.*

**Version**	**Link to animated video**
Factual information	https://vimeo.com/344270276/7cc942fa8d
Process narrative information	https://vimeo.com/345678674/e468b774c9
Experience narrative information	https://vimeo.com/344265544/5d7e01c9fc
