# Supplementary material for: Testing the effects on information use by older versus younger women of modality and narration style in a hospital report card
Source: Health Expect. 2021 Dec 24;25(2):567–78. doi: 10.1111/hex.13389 (PMC8957735; doi:10.1111/hex.13389)
Supplement: Supplementary file 3 — Supplementary information. [file HEX-25--s001.pdf]

Appendix C. *Recall questions in questionnaire*

The following questions concern the content of the information you have seen. Some questions are similar to each other. It is still important that you try to fill in all questions carefully and as correctly as possible. It is important that you fill in <u>all</u> questions. If you know the answer to a question, you can describe your answer by clicking on 'This information has been provided, namely …'

1. What have you just seen information about?
   - ○ This information has not been provided
   - ○ This information has been provided, but I do not remember
   - ○ This information has been provided, namely …

2. What is the aim of the Monitor Borstkankerzorg?
   - ○ This information has not been provided
   - ○ This information has been provided, but I do not remember
   - ○ This information has been provided, namely …

3. The Monitor Borstkankerzorg provides different TYPES of information about the quality of healthcare. How many types of information does the Monitor Borstkankerzorg contain?
   - ○ This information has not been provided
   - ○ This information has been provided, but I do not remember
   - ○ This information has been provided, namely …

4. Which three types of information about the quality of healthcare does the Monitor Borstkankerzorg provide?
   - ○ This information has not been provided
   - ○ This information has been provided, but I do not remember
   - ○ This information has been provided, namely …

5. What does information about the <u>organization of care</u> entail?
   - ○ This information has not been provided
   - ○ This information has been provided, but I do not remember
   - ○ This information has been provided, namely …

6. What examples of information about the <u>organization of care</u> were depicted in the Monitor Borstkankerzorg?
   - ○ This information has not been provided
   - ○ This information has been provided, but I do not remember
   - ○ This information has been provided, namely …

7. What does information about the <u>care process</u> entail?
   - ○ This information has not been provided
   - ○ This information has been provided, but I do not remember
   - ○ This information has been provided, namely …

8. What examples of information about the care process were depicted in the Monitor Borstkankerzorg?

- This information has not been provided
- This information has been provided, but I do not remember
- This information has been provided, namely …

9. What does information about the result of care entail?

- This information has not been provided
- This information has been provided, but I do not remember
- This information has been provided, namely …

10. What examples of information about the result of care were depicted in the Monitor Borstkankerzorg?

- This information has not been provided
- This information has been provided, but I do not remember
- This information has been provided, namely …

11. Which four hospitals does the Monitor Borstkankerzorg provide information about?

- This information has not been provided
- This information has been provided, but I do not remember
- This information has been provided, namely …

12. In which hospital(s) (MC Oost, Noordhaven Ziekenhuis, St Nathaniel of IJssel MC) is it possible to have a breast amputation and breast reconstruction simultaneously?

- This information has not been provided
- This information has been provided, but I do not remember
- This information has been provided, namely …

13. In which hospital(s) (MC Oost, Noordhaven Ziekenhuis, St Nathaniel of IJssel MC) is the waiting time between diagnosis and treatment the longest?

- This information has not been provided
- This information has been provided, but I do not remember
- This information has been provided, namely …

14. Which hospital (MC Oost, Noordhaven Ziekenhuis, St Nathaniel of IJssel MC) were patients less satisfied with?

- This information has not been provided
- This information has been provided, but I do not remember
- This information has been provided, namely …
