# Supplementary material for: Testing the effects on information use by older versus younger women of modality and narration style in a hospital report card
Source: Health Expect. 2021 Dec 24;25(2):567–78. doi: 10.1111/hex.13389 (PMC8957735; doi:10.1111/hex.13389)
Supplement: Supplementary file 4 — Supplementary information. [file HEX-25--s002.pdf]

Appendix D. *F-test statistics per research question.*

	**Effects of modality**	**Interaction effect of modality * age**	**Effects of narration style**	**Interaction effects of narration style * age**	**Effects of modality * narration style**	**Interaction effects of modality * narration style * age**
Perceived cognitive load	$F(1,629)$=1.34, p=.248	$F(1,627)$=0.36, p=.548	$F(1,628)$=0.83, p=.438	$F(2,265)$=1.72, p=.180	$F(2,625)$=0.84, p=.431	$F(2,619)$=0.56, p=.572
Satisfaction with information	$F(1,629)$=11.16, p=.001	$F(1,627)$=0.15, p=.696	$F(1,628)$=0.78, p=.460	$F(2,265)$=3.68, p=.026	$F(2,625)$=0.94, p=.392	$F(2,619)$=2.83, p=.060
Attractiveness	$F(1,629)$=17.86, $p < .001$	*	*	$F(2,265)$=2.12, p=.121	*	$F(2,619)$=1.62, p=.200
Comprehensibility	$F(1,629)$=3.21, p=.074	*	*	$F(2,265)$=1.50, p=.223	*	$F(2,619)$=3.71, p=.025
Emotional Support	$F(1,629)$=4.87, p=.028	*	*	$F(2,265)$=4.84, p=.008	*	$F(2,619)$=2.58, p=.077
Information comprehension	$F(1,629)$=4.16, p=.042	$F(1,627)$=4.00, p=.046	$F(1,628)$=1.38, p=.253	$F(2,265)$=2.02, p=.133	$F(2,625)$=0.28, p=.755	$F(2,619)$=0.32, p=.726
Information recall	$F(1,629)$=0.01, p=.946	$F(1,627)$=2.00, p=.157	$F(1,628)$=0.36, p=.697	$F(2,625)$=0.37, p=.693	$F(2,625)$=1.44, p=.239	$F(2,619)$=1.34, p=.262
Decisional Conflict	$F(1,629)$=1.78, p=.183	$F(1,627)$=0.13, p=.716	$F(1,628)$=0.54, p=.585	$F(2,265)$=1.13, p=.323	$F(2,625)$=1.21, p=.300	$F(2,619)$=1.09, p=.338
Informed	*	*	*	*	*	*
Values clarity	*	*	*	*	*	*
Support	*	*	*	*	*	*
Uncertainty	*	*	*	*	*	*
Effective decision	*	*	*	*	*	*

** If no statistically significant effect was found for both the total scale and its subscales, only the F-statistics of the total scale was reported.*
